# Supplementary material for: Identification of photoperiod-induced specific miRNAs in the adrenal glands of Sunite sheep (Ovis aries)
Source: Front Vet Sci. 2022 Jul 22;9:888207. doi: 10.3389/fvets.2022.888207 (PMC9354845; doi:10.3389/fvets.2022.888207)
Supplement: Supplementary file 1 [file Data_Sheet_1.ZIP › Supplementary file/miRDeep2 file.zip!/miRDeep2 file/Know_Structure/SPLP42A1/oar-mir-25.pdf]

miRBase precursor : oar-mir-25
Total read count : 16933
oar-miR-25 read count : 16860
remaining reads : 73

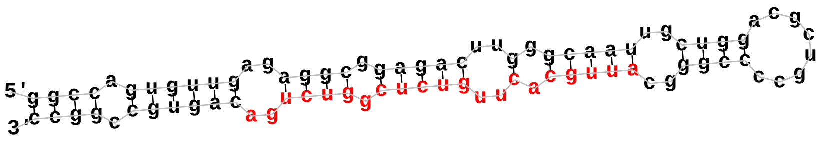

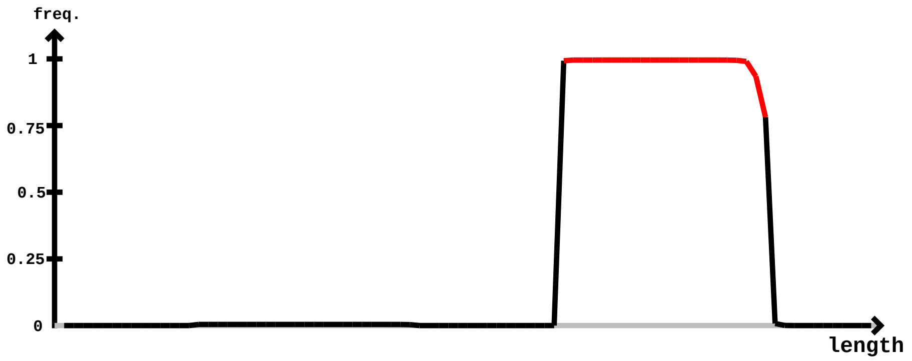

oar-miR-25

```
5'- ggccaguguugagaggcggagacuugggcaauugcuggacgcugccccgggcauugcacuugucucggucugacagugccggcc   -3'    exp
    ((((.((((((..((((.(((((..(.(((((..((((........))))..))))).)..))))).)))).)))))).))))    reads      mm         sample
    .............aggcggagacuugggcaauu...................................................    2          0          seq
    .............aggcggagacuugggcaauugc.................................................    11         0          seq
    .............aggcggagacuugggcaauugcu................................................    60         0          seq
    ..................................................gcauugcacuugucucggucuga...........    1          0          seq
    ...................................................cauugcacuugucucgg................    4          0          seq
    ...................................................cauugcacuugucucggu...............    15         0          seq
    ...................................................cauugcacuugucucgguc..............    68         0          seq
    ...................................................cauugcacuugucucggucu.............    944        0          seq
    ...................................................cauugcacuugucucggucug............    2587       0          seq
    ...................................................cauugcacuugucucggucuga...........    13067      0          seq
    ...................................................cauugcacuugucucggucugac..........    123        0          seq
    ...................................................cauugcacuugucucggucugaca.........    8          0          seq
    ....................................................auugcacuugucucggucu.............    1          0          seq
    ....................................................auugcacuugucucggucug............    12         0          seq
    ....................................................auugcacuugucucggucuga...........    29         0          seq
    .......................................................gcacuugucucggucuga...........    1          0          seq
```
